# Quantum Optics in Nanostructures

**DOI:** 10.3390/nano11081919

**Published:** 2021-07-26

**Authors:** Yulia V. Vladimirova, Victor N. Zadkov

**Affiliations:** 1 Department of Physics and Quantum Technology Centre, Lomonosov Moscow State University, 119991 Moscow, Russia; 2 Faculty of Physics, Higher School of Economics, Old Basmannya 21/4, 105066 Moscow, Russia; zadkov@isan.troitsk.ru; 3 Institute of Spectroscopy of the Russian Academy of Sciences, Fizicheskaya Str. 5, Troitsk, 108840 Moscow, Russia

**Keywords:** nanophotonics, nanoplasmonics, nanostructure, quantum emitter, two-level system, quantum optics, resonance fluorescence, antibunching and quantum statistics of photons, squeezed states, quantum entangled states

## Abstract

This review is devoted to the study of effects of quantum optics in nanostructures. The mechanisms by which the rates of radiative and nonradiative decay are modified are considered in the model of a two-level quantum emitter (QE) near a plasmonic nanoparticle (NP). The distributions of the intensity and polarization of the near field around an NP are analyzed, which substantially depend on the polarization of the external field and parameters of plasmon resonances of the NP. The effects of quantum optics in the system NP + QE plus external laser field are analyzed—modification of the resonance fluorescence spectrum of a QE in the near field, bunching/antibunching phenomena, quantum statistics of photons in the spectrum, formation of squeezed states of light, and quantum entangled states in these systems.

## 1. Introduction

The nonclassical behavior of light manifests itself in the resonance fluorescence of a single atom [1] as a sub-Poissonian distribution of the statistics of photons [2] and the phenomenon of antibunching of scattered photons [3] upon counting delayed coincidences, in the distribution of photocounts, and in the spectrum of intensity fluctuations. Over the recent decades, a high number of experiments on the study of nonclassical light from quantum emitter (QE) (atom, molecule, quantum dot, nitrogen vacancy (NV) center, etc.) has also been carried out in the developing field of nanophotonics [4].

This review is devoted to the study of effects of quantum optics of a QE in the near field of nanostructures. In the scientific literature, the term “quantum plasmonics” is also widely used, which is considered to mean a new area of research in nanophotonics—a combination of nanoplasmonics with quantum optics [5]. In this case, the interaction of QEs not only with plasmons localized inside nanoparticles (NPs) is considered, but also with surface plasmonic waves, when their quantum properties are also important.

**Figure 1:**
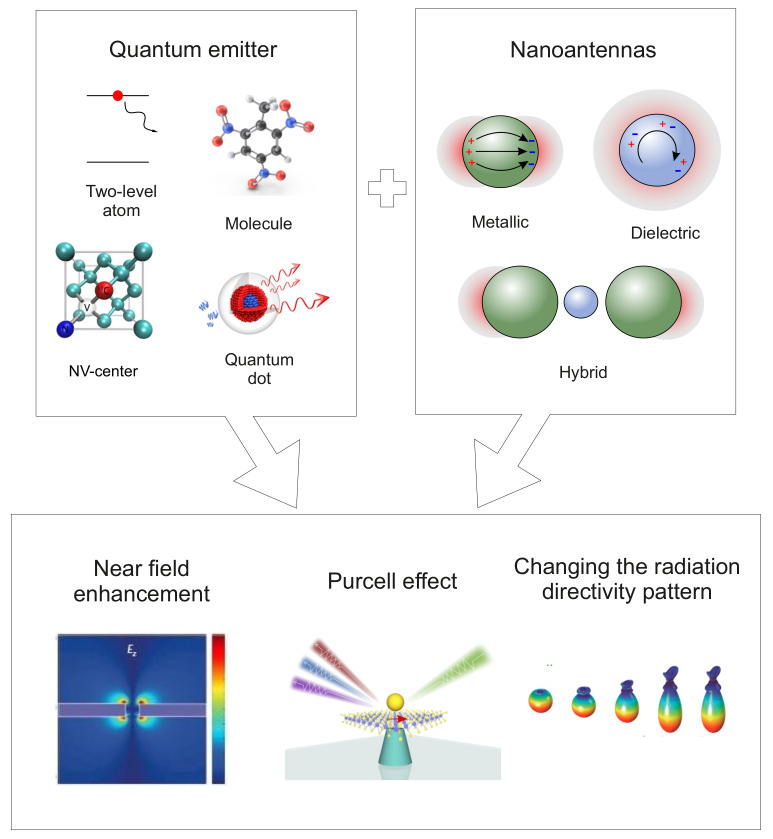
Properties of nanoantennas for controlling the electromagnetic field at the nanoscale and changes in the parameters of spontaneous radiation of a QE in the near field of a nanoantenna.

In this review, we will consider the following unique properties of NPs and nanostructures (Figure 1):

1. Efficient conversion of electromagnetic radiation that interacts with NPs into the near field. In this case, a multiple increase in the field intensity in subwavelength regions around NPs is achieved [6,7,8];
2. Controlling the parameters of spontaneous emission of a QE in the near field of an NP (Purcell effect) [9];
3. The ability to concentrate and redirect incident radiation to a given area of space—radiation directivity pattern control [10].

Nanostructures with these properties are often called optical nanoantennas. They have high potential in solving problems of optical information processing [11,12,13], in creation of single-photon sources [14], in photovoltaics [15,16,17,18,19], in medicine upon treatment of oncological diseases [20,21,22,23,24], in problems of microscopy with super-resolution [25] and many others. They are considered in a large number of reviews [26,27,28,29,30] and books [31,32,33], but here, we will only briefly dwell on the main properties of nanoantennas, which are important in describing the interaction of QEs with NPs.

Optical nanoantennas can be divided into metallic, dielectric, and hybrid (metal– dielectric) ones, each type having its own merits and drawbacks.

Metallic or plasmonic nanoantennas make it possible to localize the exciting electromagnetic field in the near field on a scale of ∼10 nm due to the excitation of plasmon resonances in them. Traditionally, these antennas are made of chemically stable noble metals, e.g., gold or silver. Even though their dissipative losses in the visible range are the smallest among metals, they are still rather high [34,35]. The geometry of a nanoantenna is also important, because it significantly affects the near-field gain and the Purcell factor, the enhancement of a quantum system’s spontaneous rate by its environment. Thus, it was shown in [36] that a solution of gold nanospheres with diameters of 35 nm does not fluoresce, whereas a solution of gold nanorods of the same volume leads to an increase in fluorescence by six to seven orders of magnitude. The ability of metal nanoantennas to enhance the near field by orders of magnitude plays an important role in thermoplasmonics and medicine [37], but their use in systems that require efficient transmission of electromagnetic energy is of little efficiency.

Dielectric nanoantennas, which are made of materials with a high refractive index and low absorption coefficient in the visible range (e.g., Si), were devised to surmount limitations of metallic nanoantennas [38]. Both electric and magnetic Mie resonances can be excited in them [39,40,41], which allow nanoantennas to equally interact with the electric and magnetic components of the electromagnetic field. This contrasts to plasmonic antennas, which interact only with the electric component. However, the gain of the near field of these antennas is markedly weaker than that of plasmonic ones, and, as a consequence, the Purcell factor is smaller.

The advantages and disadvantages of metal and dielectric nanoantennas listed above initiated the creation of the so-called hybrid nanoantennas, which combine antennas of the two previous types [28]. Studies showed that they possess many properties that are interesting for practical applications, including low thermal losses, optical and magnetic resonances response, strong nonlinear optical properties, and control of the radiation directivity pattern.

A nanoantenna of any type located near a QE affects its characteristics: radiation intensity, quantum yield, resonance fluorescence spectrum, statistics of photons from the resonance fluorescence spectrum; it leads to antibunching of photons and radiation squeezing, as well as to the generation of quantum entangled states. All of these properties depend on the strength and polarization of the electromagnetic field, the rate of spontaneous relaxation, and the transition frequency of the QE [42].

In Section 2 of this review, we will consider the theoretical description of the distributions of the strength and polarization of the near field of a plasmonic NP, as well as the dependences of the spontaneous relaxation rate and the shift of the resonance transition frequency of a QE on its position in the near field of the NP.

Section 3 of the review will be devoted to the effects of interaction of a QE with a nanoantenna. The quantum efficiency of the nanoantenna in the classical and quantum approaches, the possibility of controlling the parameters of the QE by changing the NP parameters, and fluorescence quenching of the QE near the nanoantenna will be considered. The case of hybrid nanoantennas will be considered separately.

Section 4 of the review will consider the effects of quantum optics of a QE in the near field of an NP in an external electromagnetic field. The generation of the resonance fluorescence spectrum of the QE, the phenomenon of photon antibunching, the formation of squeezed states of light, the sub-Poissonian statistics of photon counting from the resonance fluorescence spectrum, and the generation of quantum entangled states will be considered.

In Conclusions, the main effects of quantum optics of a QE in the near field of an NP will be summarized and issues referring to their applications will be discussed.

## 2. The Near Field of an NP in an External Electromagnetic Field

The simplest geometry of NPs is spherical. We will begin our consideration with such NPs because they admit an analytical solution of the diffraction problem, which was first obtained by Gustav Mie in 1908 [39]. The Mie theory as applied to spherical NPs has been considered in detail in the literature [28,33,43]. Further development of the Mie theory made it possible to analytically calculate the optical properties of NPs of spheroidal geometry (nanorods, nanoneedles, nanodiscs). These problems require efficient computation of spheroidal functions using efficient algorithms [44,45]. In the case of complex geometries, the only way to find a solution is to numerically solve Maxwell’s equations, and a whole number of efficient numerical methods have also been developed for this purpose.

However, is not always optimal to directly solve Maxwell’s equations. For example, the numerical solution of the Maxwell equations for the scattering of the electromagnetic field by NPs made of one material cannot be used to determine the plasmonic properties of NPs of the same shape but made from a different material. Finding the exact values of plasmon frequencies and other characteristics of plasmons with this solution method is a difficult problem. Instead, it is convenient to use the so-called ε method, in which the main role is played not by the resonance frequencies, but rather by the corresponding values of the dielectric permittivity.

This method was developed in [46,47], and it allows one to find the near field of an NP from its plasmonic spectrum, i.e., eigenfunctions $e_{n}$ and $h_{n}$, satisfying Maxwell’s equations

(1) $$\mathrm{rot}h_{n}+ik\varepsilon_{n}e_{n}=0,\;\mathrm{rot}e_{n}-ikh_{n}=0.$$

The continuity of tangential components on the particle surface and the Sommerfeld radiation conditions should be also provided. As a result, the electric field near an NP of an arbitrary shape is defined as

(2) $$E\left(r,\omega \right)=E_{0}\left(r\right)+\sum_{n}e_{n}\frac{\varepsilon \left(\omega \right)-\varepsilon_{H}}{\varepsilon_{n}-\varepsilon \left(\omega \right)}\frac{\int_{V}\left(e_{n},E_{0}\right)dV}{\int_{V}e_{n}^{2}dV},$$

where ε(ω) is the dependence of the dielectric permittivity of the specific material on the frequency, $e_{n}$ and $\varepsilon_{n}$ are the modes of plasmonic oscillations and the eigenvalue that corresponds to the given mode and $E_{0}$ and *V* are the field in the absence of the NP and the volume of the NP. A similar expression is obtained for H(r,ω) up to the replacement $e_{n}\to h_{n}$, $E_{0}\to H_{0}$. Equation (2) is accurate and describes an NP of any size, because it completely takes into account the effect of retardation.

In our review, we are dealing with optical fields in the visible range, when the wavelength of the external field greatly exceeds the particle size λ≫l. In this case, the quasi-static approximation works well for determining near fields $e_{n}$. In this case, the field near an NP, according to (2), is described by the formula

(3) $$E=E_{0}+\sum_{n}\frac{\varepsilon \left(\omega \right)-\varepsilon_{H}}{\varepsilon_{n}-\varepsilon \left(\omega \right)}\frac{\int_{V}\left(-\nabla \varphi_{n},E_{0}\right)dV}{\int_{V}\nabla \varphi_{n}^{2}dV}\left(-\nabla \varphi_{n}\right),$$

where $\varphi_{n}$ represents the potentials of the corresponding plasmonic modes $e_{n}$.

### Polarization of the Near Field of an NP

In problems of nano-optics and nanoplasmonics, the interaction of a QE with the field depends crucially on the polarization of the latter. To describe the polarization, the so-called generalized Stokes parameters are commonly used [48]. In the three-dimensional case, their number is nine, and three of them play a role similar to parameter $S_{3}$ for each of the three planes xy, yz, xz and determine the predominance of the right-hand circularly polarized field component over the left one. The expressions for these parameters have the form

(4) $$\begin{array}{c} S_{3xy}=i\left[\langle E_{x}^{*}\left(r,\omega \right)E_{y}\left(r,\omega \right)\rangle -\langle E_{x}\left(r,\omega \right)E_{y}^{*}\left(r,\omega \right)\rangle \right],\\ S_{3xz}=i\left[\langle E_{x}^{*}\left(r,\omega \right)E_{z}\left(r,\omega \right)\rangle -\langle E_{x}\left(r,\omega \right)E_{z}^{*}\left(r,\omega \right)\rangle \right],\\ S_{3yz}=i\left[\langle E_{y}^{*}\left(r,\omega \right)E_{z}\left(r,\omega \right)\rangle -\langle E_{y}\left(r,\omega \right)E_{z}^{*}\left(r,\omega \right)\rangle \right], \end{array}$$

where $E_{i}=A_{i}\exp\left(i\omega t\right)$, $A_{i}$, and the angle brackets denote time averaging. In our case, the fields are stationary; therefore, the operation of averaging can be omitted. Parameter $S_{3}$ takes negative values if the left-hand circular polarization predominates and its values are positive if the right-hand circular polarization prevails.

In terms of the three-dimensional Stokes parameters, the degree of polarization can be written as

(5) $$P=\sqrt{1-{\left(\frac{S_{3xy}}{I}\right)}^{2}-{\left(\frac{S_{3xz}}{I}\right)}^{2}-{\left(\frac{S_{3yz}}{I}\right)}^{2}},$$

where, for convenience, the Stokes parameters are normalized to the intensity of the field at the point of observation.

The authors of [49,50] examined the polarization of the near field of a spheroidal silver NP that was excited by a plane wave of linear, circular, or elliptical polarization. If an NP interacts with a plane electromagnetic wave, only dipole modes are excited in it.

**Figure 2:**
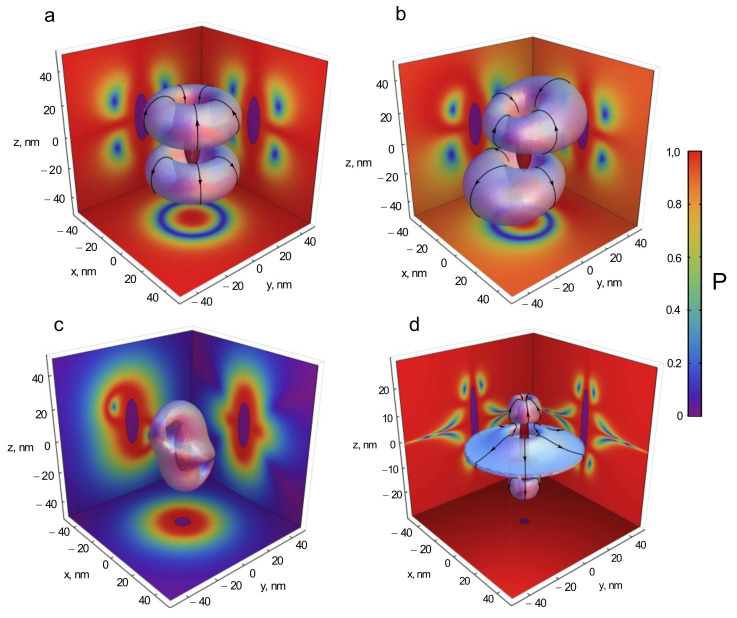
Distributions of the degree of polarization of the near field upon incidence of an external field that is linearly polarized along the *z* axis ($E_{0,x}=0$ V/m, $E_{0,y}=0$ V/m, $E_{0,z}=200$ V/m) in the case of a plasmon resonance $\alpha =\alpha_{\mathrm{res}}$. The three-dimensional surface corresponds to a value of P=0.8. The two-dimensional distributions describe the cross sections in three planes: xz, yz, and *z* = 15 nm (in cases (**a**,**b**)) and xy (in cases (**c**,**d**)). The red color corresponds to the linear polarization (P=1), while the violet color corresponds to the circular polarization (P=0). The direction of rotation of the vector *E* is shown by arrows.

The calculation results in Figure 2 show that the degree of the polarization distribution pattern substantially depends on the polarization of the external field and has a complex structure. Regions with polarization opposite to the polarization of the external field arise near the NP surface; if the external field is linearly polarized, the distribution of the degree of polarization has a shape of two symmetric tori, in the center of which the polarization is circular. Conversely, if the polarization of the external field is circular, the distribution of the degree of polarization has the shape of a complex figure, in the center of which the polarization is linear. If the external field is linearly polarized, the regions of polarization change decrease and shift towards the nanoantenna tips at $\alpha <\alpha_{\mathrm{res}}$ and towards the equator of the spheroid at $\alpha >\alpha_{\mathrm{res}}$. It is important to note that the positions of the regions can be controlled by changing the direction of incidence of the external radiation.

In [51], the polarization of the near field in the same system was studied using the isotropy parameters $L^{T}$ and $C^{T}$, which characterize the distribution of ellipses near singular points. Numerical algorithm that tracks $L^{T}$ and $C^{T}$ lines makes it possible to visualize the lines of polarization singularities near the nanospheroid.

It is important to note that a large number of theoretical studies are devoted to the study of the properties of NPs in free space, although NPs in real experiments are surrounded by other objects, e.g., by other NPs or the substrate on which they are deposited. In some cases, the influence of the surrounding medium can be neglected; however, this simplification is not always valid. The influence of the substrate can be quite significant, for example, in the development of near-field sensors, as well as in the popular method of surface-enhanced Raman scattering (SERS) [52].

Thus, in [53], the distribution of the polarization of the near field near a silver nanospheroid on a quartz substrate was investigated. It was found that, as in the case of the absence of a substrate, the polarization of the near field near the surface of the spheroid is reversed. However, whereas the distribution of the degree of polarization of NPs in free space is symmetric with respect to both the major and the minor axes of the spheroid, in the presence of a substrate, regions of the polarization change in the substrate are observed only for wavelengths near the plasmon resonance, and the size of these regions inside the substrate decreases. For example, at exact resonance, the size of the region of opposite polarization inside the substrate decreases by approximately half as compared to the region above the substrate. Far from the plasmon resonance, there are no regions of opposite polarization in the substrate.

The near-field polarization was also studied for a dielectric nanosphere with a high refractive index (Si) both in free space and on a quartz substrate [54,55]. In the case of a dielectric NP, regions of change in the polarization are most pronounced at wavelengths that correspond to electric and magnetic dipole and quadrupole resonances. As in the case of a metallic NP, regions of polarization reversal are formed near the NP surface; however, the shape of these regions changes. In [56], the polarization of the near field for a dielectric nanosphere with a high refractive index (Si) in free space was studied using the isotropy parameters $L^{T}$ and $C^{T}$.

## 3. Spontaneous Emission of Radiation of a QE Near an NP

With the invention of lasers in 1960, quantum electronics has been actively developing in the direction of using controlled stimulated quantum processes to improve all characteristics of laser radiation. Forty years later, due to the development of nanotechnologies, controlling elementary spontaneous emission process of a QE began to be actively investigated, which formed the basis for a wide spectrum of applications, such as LEDs on heterostructures [57], analytical chemistry, spectroscopy, sensors [58], etc.

A fluorescent QE is an ideal choice as a label in many technologies, such as gene sequencing [59,60], neural mapping [61], monitoring of safety of food and drugs [62] and many others. At the same time, the spontaneous emission of a single QE in free space is too weak to be detected at low concentrations of QEs. In addition, because the radiation directivity pattern of such a QE does not have any preferred direction, there is a problem of efficient signal registration. To combat these disadvantages, methods of increasing the fluorescence intensity and the radiation directivity pattern control are needed.

The question of controlling the spontaneous emission of radiation by a QE was posed for the first time by Purcell [9], who considered an increase in the probability of spontaneous emission of an atom in a cavity as the frequency of the only one mode of which was tuned to the transition frequency of the atom. In his work, Purcell showed that the rate of spontaneous relaxation of a magnetic dipole in a resonator can increase in comparison with the relaxation rate in free space—i.e., the surrounding medium of the atom significantly changes its radiative properties. Nanocavities, as Purcell considered, and nanoantennas can act as nanoenvironments.

Purcell’s formula for a single-mode cavity states that the rate of emission of a fluorophore in a dielectric resonator increases as compared to that in a vacuum with the coefficient

(6) $$F=\frac{3}{4\pi^{2}}\lambda^{3}\frac{Q}{V},$$

where *Q* is the quality factor of the cavity mode and $V/\lambda^{3}$ is the volume of the mode in cubic wavelengths.

Microcavities can maintain modes of an extremely small volume, which immediately leads to the requirement for large values of *Q* ($Q>10^{4}$). Therefore, the larger the Purcell factor for the nanocavity we want to obtain, the narrower will be the linewidth and the more complicated the cavity tuning process for a particular QE. In addition, a high *Q* value implies slow (picosecond or nanosecond) response times, which can ultimately interfere with ultrafast switching. In contrast to nanocavities, nanoantennas are broadband open systems with typical values of *Q* lying in the range of 3÷30. An increase in the emission rate can occur over the entire QE spectrum of, e.g., an organic dye or a quantum dot. Thus, the need to obtain a small mode volume requires storing electromagnetic energy in material resonance. Therefore, single-photon nanoantennas are made of plasmonic or surface-plasmon-polariton materials, and recently, due to the development of technologies, they have been made of hybrid (metal–dielectric) materials [63].

We note that quantities *Q* and *V* in (6) are not defined for plasmonics [64,65] if the frequency is used as an eigenvalue, in this case, the eigenfunctions increase indefinitely at infinity, which leads to problems upon numerical solution of equations in partial derivatives. This review uses a more powerful approach (Section 2), where the eigenvalue is the dielectric permittivity, the eigenfunctions decay at infinity, and all quantities are well defined for any system.

Prior to discussing the modification of the spontaneous relaxation rate near nano-objects, we note that, in the theory of interaction of an atom and field, there are two clearly defined regimes: strong and weak coupling. The difference between the regimes is determined by the atom–field coupling constant

(7) $$\varkappa =d\sqrt{\frac{\hbar \omega_{0}}{2\varepsilon_{0}\varepsilon_{r}V}},$$

where $\omega_{0}$ is the frequency of the atomic transition, *d* is its dipole matrix element, $\varepsilon_{0}$ is the dielectric permittivity of a vacuum, $\varepsilon_{r}$ is the relative dielectric permittivity, *V* is the cavity volume. The condition ϰ≪γ defines the weak coupling regime, while the strong coupling regime corresponds to the condition ϰ≫γ, where γ is the photon relaxation rate in the cavity.

In the weak coupling regime, classical theory and calculations within the framework of quantum electrodynamics (QED) yield the same result for the modification of the rate of spontaneous radiative relaxation. In the strong coupling regime, the spontaneous decay of atoms, as was shown in [66,67,68,69], is also well described within the classical approach. In this case, in terms of the classical theory, the modification of the spontaneous relaxation is determined by the scattering of atomic fields by the environment, while, in the framework of QED, the relaxation is partially caused by fluctuations of the vacuum field, which, in turn, is a function of the environment.

### 3.1. Classical Approach

Let us consider spontaneous relaxation under the classical approach [70] modeling the QE by a free dipole that oscillates according to a harmonic law. In free space, the equation of motion of the dipole has the form

(8) $$\frac{d^{2}}{dt^{2}}d\left(t\right)+\gamma_{0}\frac{d}{dt}d\left(t\right)+\omega_{0}^{2}d\left(t\right)=0,$$

where d=Δr is the electric dipole moment of the oscillator and $\omega_{0}$ is its eigenfrequency. The rate of spontaneous decay in the vacuum $\gamma_{0}$ is defined as

(9) $$\gamma_{0}=\frac{1}{4\pi \varepsilon_{0}}\frac{2e^{2}\omega_{0}^{2}}{3mc^{3}}.$$

If the oscillator is located near the NP, then it is exposed to the action of an additional field $E_{s}\left(r_{0},t\right)$, and the equation of motion takes the form

(10) $$\frac{d^{2}}{dt^{2}}d\left(t\right)+\gamma_{0}\frac{d}{dt}d\left(t\right)+\omega_{0}^{2}d\left(t\right)=\frac{e^{2}}{m}E_{s}\left(r_{0},t\right).$$

To find the reflected field $E_{s}\left(r_{0}\right)$, it is necessary to solve the complete system of Maxwell’s equations, in which the source of charge and current is a dipole moment of the oscillator. The expression for the change in the rate of spontaneous decays, which is called the Purcell factor, has the form

(11) $$\frac{\gamma }{\gamma_{0}}=1+\frac{6\pi \varepsilon_{0}}{|d_{0}{|}^{2}}\frac{1}{k^{3}}ℑ\left[d_{0}\cdot E_{s}\left(r_{0},\omega_{0}\right)\right],$$

where $d_{0}$ is the dipole moment of the oscillator in vacuum. It is also applicable in the case of a complex dielectric permittivity, i.e., in the case of a material with losses.

The change in the rate of spontaneous emission of a QE in the presence of an NP was investigated for the first time in [71,72], and it was shown that the radiation part of the decay rate has the form

(12) $$\frac{\gamma }{\gamma_{0}}=\frac{|d_{\mathrm{tot}}{|}^{2}}{|d_{0}{|}^{2}},$$

where $|d_{\mathrm{tot}}{|}^{2}$ is the total dipole moment of the system QE + NP. Equations (11) and (12) can be applied to any NPs, including NPs with losses.

### 3.2. Quantum Approach

In quantum electrodynamics, to describe spontaneous relaxation, the interaction of a QE with the continuum of states is considered. In the simplest case, we will describe the QE by a two-level system with the ground state |g〉 and the excited state |e〉, $\omega_{0}$ is the transition frequency, and d is the transition dipole moment. According to Fermi’s golden rule, the spontaneous relaxation rate is defined as

(13) $$\gamma =\frac{2\pi }{\hbar }\sum_{f}\left|\langle f\right|{\hat{H}}_{I}{\left|i\rangle \right|}^{2}\delta \left(\omega_{i}-\omega_{f}\right),$$

where ${\hat{H}}_{I}=-\hat{d}\cdot \hat{E}$ is the interaction Hamiltonian in the dipole approximation. Here, we consider combined states “field + system” and transitions from the excited initial state |i〉=|e,{0}〉 with the energy $E_{i}$ to a set of final states $|f\rangle =|g,\left\{1_{\omega_{k}}\right\}\rangle$ with the same energy $E_{f}$. The final states differ from each other only with respect to mode *k* of the radiation field. The number of individual single-photon states is defined by the local density of states $\rho_{d}\left(r_{0},\omega_{0}\right)$, $r_{0}$, where $r_{0}$ defines the position of the two-level system.

The expression for the spontaneous relaxation in terms of the Green’s function has the form [31]

(14) $$\gamma =\frac{2\omega_{0}}{3\hbar \varepsilon_{0}c^{2}}{\left|d\right|}^{2}\rho_{d}\left(r_{0},\omega_{0}\right),\;\rho_{d}\left(r_{0},\omega_{0}\right)=\frac{6\omega_{0}}{\pi c^{2}}nℑG\left(r_{0},r_{0},\omega_{0}\right)n,$$

where the dipole moment is represented by the product d=dn in which n is the unit vector along the direction d. This formula makes it possible to calculate the spontaneous relaxation rate of a two-level QE in an arbitrary environment, provided that the Green’s function for the environment is known. The spontaneous relaxation rate is expressed in terms of the partial local density of states ρ, which corresponds to the number of modes per unit volume and frequency in a point quantum system with coordinate $r_{0}$ in which a photon with energy $\hbar \omega_{0}$ can be emitted during the process of spontaneous relaxation. In the literature, $\rho_{d}$ is called PLDOS (Partial Local Density of Optical States)

We note that Equation (14), for the total relaxation rate of a QE, is valid in the presence of arbitrary losses in the medium both in the classical and in the quantum formulations of the problem [73]. For NPs free of losses, the quantum mechanical approach usually makes it easier to obtain the result, since it is immediately expressed in the form of a rather simple expansion in solutions of homogeneous Maxwell equations. In [74,75,76], the procedure for quantization of electromagnetic fields was generalized to the case of dispersive and absorbing media. In terms of this theory, it was shown that, in the first order of the perturbation theory, the classical and quantum results for the rates of spontaneous transitions are equivalent.

If the surrounding medium is homogeneous and isotropic, the spontaneous relaxation rate (14) is averaged over all possible orientations, and the partial local density of states becomes identical to the total local density of states ρ (LDOS, Local Density of Optical States), defined as

(15) $$\rho \left(r_{0},\omega_{0}\right)=\frac{2\omega_{0}}{\pi c^{2}}ℑ(Tr\left(G\left(r_{0},r_{0},\omega_{0}\right)\right).$$

Experiments to study the effect of nanostructures on the spontaneous relaxation process began in the 1960s. The influence of the interface between the media on the spontaneous relaxation of molecules was experimentally verified for the first time in 1966 in [77], and then, 17 years later, it was verified in [78]. It is important to emphasize that the nanoenvironment is capable not only of increasing the relaxation rate, but also of decreasing it [79]. The inhomogeneous environment also affects the nonradiative energy transfer by adjacent molecules (the so-called Förster energy transfer [80]).

The concept of an optically excited nanoantenna with a single fluorophore as a QE was proposed for the first time in [81]. In the early 2000s, researchers used individual fluorescent molecules as a point probe to quantitatively estimate the concentration of the electromagnetic field near the tip of a scanning microscope. It was shown that the radiation directivity pattern of single molecules can be strongly modified by a metallized probe, which, in fact, acted as a plasmonic nanoantenna.

**Figure 3:**
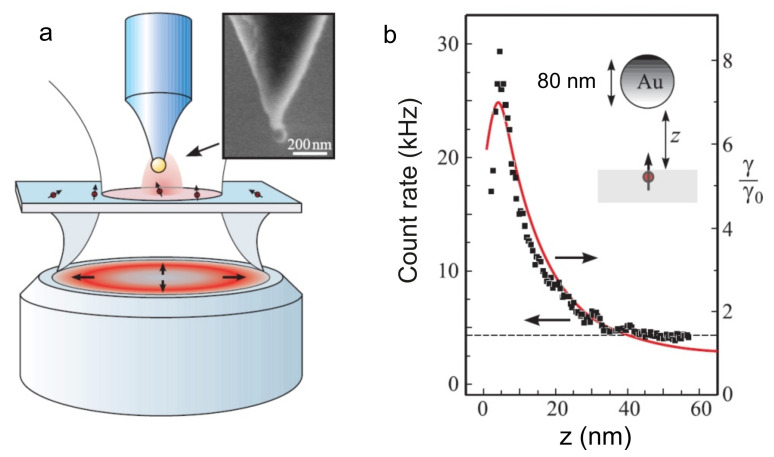
The influence of a gold NP placed at the end of a scanning microscope tip on the fluorescence of an individual Nile blue dye molecule: (**a**) schematic of the experimental setup, (**b**) experimental (dots) and theoretical (solid curves) dependences of the fluorescence intensity on the distance between the nanoantenna and the dye molecule. Adapted with permission from [82]. © 2021 American Physical Society.

In 2006, two independent research groups investigated in their works [82,83] a change in the fluorescence of a Nile blue dye molecule in relation to the distance to a gold nanosphere with a diameter of 80 nm, which was attached to the tip of a scanning microscope. The results of measurements and theoretical calculations, presented in Figure 3, showed that, when the nanosphere approaches the molecule, the fluorescence intensity initially increases by a factor of 30, but then this increase is replaced by quenching due to the predominance of nonradiative processes.

**Figure 4:**
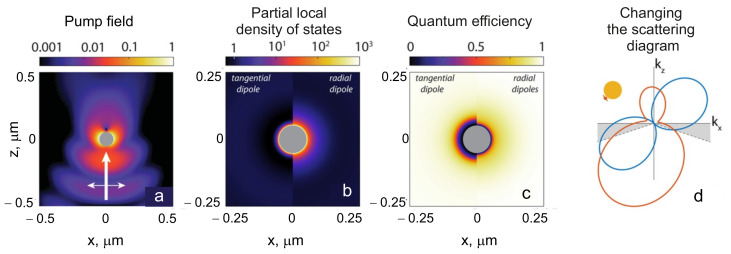
An example of a 100 nm gold Mie sphere in water that is excited by a narrow (NA = 1.3) laser beam at a wavelength of 567 nm, with the emission of the sphere occurring at a wavelength of 600 nm. (**a**) The pump field distribution (first term in (16)) for a wave that is incident from below and is polarized along the *x*-axis. (**b**) The partial local density of states near the same Mie sphere (at a radiation wavelength of 600 nm) is strongly enhanced on the metal surface, especially for radial transition dipole moments. (**c**) Quantum efficiency (second term in (16)) of the radiation under the condition that the internal efficiency of the QE strongly decreases in a shell around the metal with a radius of 10 nm. (**d**) Examples of the radiation redistributions in this case for a QE (at the top of the sketch), almost on the surface of the sphere and at a distance of 10 nm from the sphere, into the xz-plane (arbitrary geometry). Polar diagram for a free QE (blue curve) and a QE near the sphere (orange curve). The gray shaded area defines the typical acceptance cone of a high NA objective lens. Depending on the geometry, the collection probability can vary greatly (third term in (16)). The figure was taken from [85].

Experiments have clearly demonstrated three fluorescence enhancement factors, with their product determining the count rate of single fluorescent photons emitted by the QE, and each of these factors is involved in changing the electromagnetic field around the antenna:

(16) $$I\left(r,\omega_{\mathrm{pump}},\omega_{\mathrm{em}}\right)\propto P_{\mathrm{pump}}\left(r,\omega_{\mathrm{pump}}\right)\varphi \left(r,\omega_{\mathrm{em}}\right)C_{\mathrm{NA}}\left(r,\omega_{\mathrm{em}}\right),$$

where argument r underlines the dependence of the intensity on the QE location, $\omega_{\mathrm{pump}}$ is the pump frequency, $\omega_{\mathrm{em}}$ is the frequency of the fluorescent radiation, and $C_{NA}$ is the probability to detect an emitted photon by the detector. Figure 4 illustrates these factors for the case of a metallic nanosphere. We note that Equation (16) assumes that the fluorescence intensity depends linearly on pumping, which takes place only at low pump intensities [84], corresponding to the majority of experiments with single QEs.

The factor $P_{\mathrm{pump}}$ (Figure 4a) corresponds to the enhancement of the excitation of a single fluorophore and depends on the scattering properties of the antenna at the pump wavelength. As soon as the molecule is excited, the remaining factors come into the play. The collected signal depends on the quantum efficiency φ(r), i.e., on the probability that the excitation of a QE will result in the emission of a photon.

The excitation energy of any quantum system dissipates either radiatively with the emission of a photon, or nonradiatively, e.g., due to quenching by surrounding atoms or molecules. The relaxation rate is a sum of the radiative $\gamma_{r}$ and nonradiative $\gamma_{\mathrm{nr}}$ rates. In experiments to enhance fluorescence, it is necessary to ensure that either the quantum efficiency of QEs or the quantum yield $\varphi \left(r\right)=\gamma_{r}/\left(\gamma_{r}+\gamma_{\mathrm{nr}}\right)$ would be maximum. In a homogeneous medium, the quantum efficiency φ(r) is identical to the internal quantum efficiency $\varphi_{0}$, whose value lies in the range from 0 to 1, while the parameter itself determines the part of the energy losses that are associated with radiation emission. Inhomogeneities lead to changes in $\gamma_{r}$ and $\gamma_{\mathrm{nr}}$, because they are functions of the local environment. A particular medium can either increase or decrease the quantum efficiency (Figure 4c). A change in the quantum efficiency is determined by PLDOS, whose dependence on the orientation of the dipole near the gold nanosphere is shown in Figure 4b.

We note that, often, any influence of PLDOS on the spontaneous emission rate of a QE in an arbitrary nanoenvironment is usually called the Purcell factor or Purcell enhancement, although Purcell himself never considered any system other than a cavity. However, the term the Purcell enhancement has taken hold in the literature, and the terms Purcell enhancement and PLDOS are often used as equivalent to one another.

Apart from a change in the quantum efficiency, nanoantennas can also strongly change the radiation directivity pattern (Figure 4d). Creation of an antenna with a given directivity pattern leads to an increase in the harvesting efficiency of fluorescent photons by the optical system at a specific solid angle in the far zone.

The aforelisted fluorescence enhancement effects determine the main challenges to be solved when creating optically pumped single-photon nanoantennas: (i) controlling the pump gain, quantum efficiency, and directivity improvement effects to obtain compelling advantages in performance, (ii) controlling the positioning and orientation of individual QEs and antennas with nanometer accuracy, and (iii) developing the geometry of a radiating antenna to design the required radiation directivity pattern.

### 3.3. Studies of QEs Near NPs and on Substrates

In first experiments with single QEs, the lifetimes of atoms and ions, in particular, of the Eu${}^{3+}$, located near a partially reflecting plane surface were investigated [86,87]. This geometry makes it possible to perform accurate calculations within both the classical [86,88] and the quantum approaches [89]. Comparison of theoretical and experimental results showed good agreement.

A large number of works have been published on the study of spontaneous emission of QEs near NPs of specific geometries, such as spherical [90,91] and infinite circular ideally conducting cylinders [92,93,94,95,96]. In [71], simple asymptotics were obtained for an atom located on the surface (radial orientation) of a dielectric cylinder with dimensions smaller than the wavelength.

NPs of spheroidal geometry are of special interest, since the influence of a spheroid on the spontaneous emission of a QE is intermediate between the cases of a sphere and a cylinder and makes it possible to efficiently control the rate of both radiative and nonradiative decay processes of the excited state of the QE. Controlling the geometric dimensions of the nanospheroid by the ratio of the semiaxes b/a allows one to “tune” to the plasmon resonances of a particular substance.

A significant acceleration of spontaneous decays near a nanospheroid in the case of plasmon resonance can be used to underlie the operation of an apertureless scanning microscope with a single molecule as an object. In such a microscope, the tip can be approximated by an elongated nanospheroid, in which plasmon resonance can be excited at the emission frequency of the molecule. In this case, the absorption band of the molecule is assumed to be located far from the plasmon resonance. Calculations that were performed in [97,98] showed that in a microscope of this kind both the position of the molecule and the orientation of its dipole moment can be determined with a nanometer resolution.

Another model of the microscope tip can be represented by a conical surface. Theoretical calculations of the spontaneous decay rate of a QE near this surface were carried out in [99,100].

**Figure 5:**
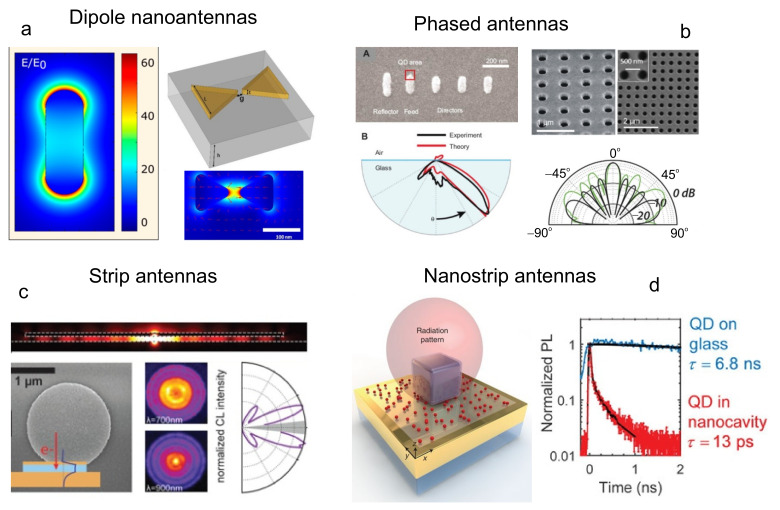
Single-photon nanoantennas: (**a**) Dipole antennas in the shape of nanorods and dimer/slot antennas. They have been reported to give a 1000-fold increase in the fluorescence brightness for intrinsically poor emitters in equal parts by pumping and improving LDOS. (**b**) A phased NP or antennas with nanoholes change the radiation directivity, usually, with a poor Purcell factor control. (**c**) Strip antennas are metal–insulator–metal waveguides for obtaining high LDOS. Radiation leakage from the edges is directional and depends on the size of the area. (**d**) Strip nanoantennas on the basis of a metallic NP, a dielectric, and a metal; according to the literature, they show more than 500-fold increase by Purcell and almost 2000-fold increase in brightness for really good emitters. However, although this antenna is directional to some extent, it is difficult to be controlled.

There are a large number of plasmonic nanoantennas, which can significantly enhance the near field, but will not lead to an efficient increase in the QE fluorescence. Here, the definition of dark plasmonics, when nonradiative decay channels dominate, and bright plasmonics, when the radiative decay channel dominates, will be intuitively comprehensible. In [85], a classification of the types of antennas that yield bright plasmon-enhanced radiation is given (Figure 5).

For dipole antennas (Figure 5a), such as nanorods [101] and dimeric antennas with gaps [102,103,104] the enhancement of up to 1000-fold has been reported, with the fluorescence brightness being increased in equal proportions due to the pumping gain and LDOS. A detailed description of the spontaneous emission of a QE near dipole nanoantennas of the most general shape (nanoellipsoids) was given in [105,106], and that near disks in [27,107,108].

An array of phased NPs [109] or an antenna in the form of a group of nanoholes [110,111] (Figure 5b) allows one to control the radiation directivity, but, as a rule, the Purcell factor for such antennas is rather low. Stripline antennas [112,113] (Figure 5c) provide high LDOS. Radiation through the edges is directional, depending on the size of inclusions.

Nanocavities integrated with quantum dots (QDs) [114 ] lead to an increase in the total radiation intensity by a factor of about $2\times 10^{3}$. They have a universal geometry, into which many other QEs can be incorporated, such as, e.g., NV (Nitrogen Vacancy) centers.

At frequencies near multipole resonances, dielectric antennas also make it possible to obtain a pronounced directivity [115,116,117] and can yield a significant increase in the fluorescence brightness [118]; however, a strong increase in the Purcell factor is much more difficult to achieve here [119].

At the present time, the so-called hybrid nanoantennas are being studied more and more intensely. In [104], the concept of a hybrid metal–-dielectric nanoantenna of a “bowtie” type was proposed. This nanoantenna is a conventional plasmonic nanoantenna in the shape of a bowtie, whose ends are made of diamond containing a nitrogen-substituted vacancy (NV center). The authors of [104] showed that the mode volume in such a nanoantenna is very small, and the electric field is concentrated in the center of the bowtie, where NV centers are concentrated. This increases the Purcell factor to values on the order of 110, and, also, increases the collection efficiency of emitted photons by a factor of 1.77.

## 4. Quantum Optics of a QE in the Near Field of an NP

Here, we will consider the effects of quantum optics in the system of a QE in the near field of a plasmonic NP in an external electromagnetic field. The mechanisms by which the rates of radiative and nonradiative decay in a simplest model of a two-level QE located in an immediate vicinity of a plasmonic NP are modified, as well as the distributions of the intensity and polarization of the near field around the NP, were considered in detail in Section 2. Here, we will consider the effects of quantum optics in the system “(NP + QE) + external laser field”: modification of the resonance fluorescence spectrum of the QE in the near field, the phenomenon of bunching/antibunching and quantum statistics of photons in the spectrum, formation of squeezed states of light, and generation of quantum entangled states in such systems.

### 4.1. Spectrum of Resonance Fluorescence of QEs

We analyzed the influence of an NP on the resonance fluorescence spectrum of a two-level QE by considering a spherical plasmonic NP. We considered the spectrum of resonance fluorescence, rather than, e.g., the spectrum of spontaneous fluorescence, since this spectrum carries information about the quantum properties of the interaction of light with QE + NP. This problem was solved for the first time in [120]. A two-level QE is located near an NP at a point with a radius vector $r_{0}$, and the QE + NP system interacts with a linearly polarized laser field whose linewidth is $\Delta \omega_{L}=1$.

**Figure 6:**
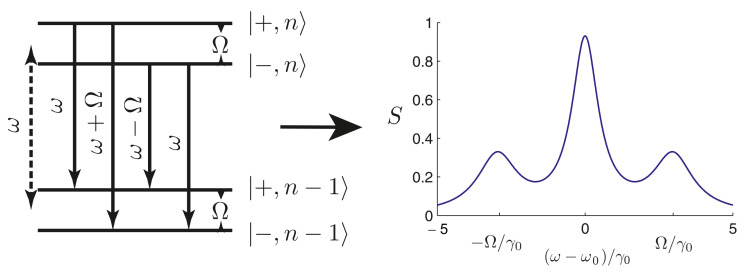
Energy level diagram of split levels of a two-level QE (“dressed” states) and resonance fluorescence spectra of a QE in a strong field in the absence of an NP ($\Omega >\gamma_{0}$) (Apanasevich–Mollow triplet). In this case, index *n* denotes the energy of the level expressed in photons of the field mode ω.

The resonance fluorescence spectrum of a two-level QE in free space consists of three well separated spectral lines of the Apanasevich–Mollow fluorescence triplet, which was first predicted by P. A. Apanasevich in [121,122], and then, by Newstein [123] and Mollow [124]. Experimentally, resonance fluorescence was studied in detail in [125]. Figure 6 shows the structure of energy levels of a two-level QE (“dressed states”) (left panel) and the spectrum of its resonance fluorescence (right panel).

The theory of resonance fluorescence of a single QE is well known [126]. This approach can be applied to the case of a QE in any surrounding medium. It follows from the theory that the spectral density of the fluorescence radiation emitted by a QE (resonance fluorescence) is determined by the normally ordered correlation function $\langle E^{\left(-\right)}\left(r,t\right)E^{\left(+\right)}\left(r,t+\tau \right)\rangle$ of the fluorescent light at some specific selected point r in the far-field zone, where $E^{\left(+\right)}\left(r,t\right)$ and $E^{\left(-\right)}\left(r,t\right)$ are the positive and negative frequency parts of the electric field operator:

$$S\left(r,\omega_{L}\right)=ℜ\int_{0}^{\infty }d\tau \langle E^{\left(-\right)}\left(r,t\right)E^{\left(+\right)}\left(r,t+\tau \right)\rangle e^{i\omega_{L}\tau }.$$

For a two-level QE, the spectral density S(r,L) of the electromagnetic field at point r takes the following form [126]:

(17) $$\begin{array}{c} S\left(r,\omega_{L}\right)=I_{0}\left(r\right)\sin^{2}\psi \left(\frac{\Omega^{2}\left(r\right)}{\gamma^{2}\left(r\right)+2\Omega^{2}\left(r\right)}\right)\\ \times \left[\frac{\gamma^{2}\left(r\right)}{\gamma^{2}\left(r\right)+2\Omega^{2}\left(r\right)}\delta \left(\omega -\omega_{L}\right)+\frac{\gamma (r)}{{\left(\omega -\omega_{L}\right)}^{2}}+\frac{\alpha_{+}\left(r\right)}{{\left(\omega +\mu \left(r\right)-\omega_{L}\right)}^{2}}+\frac{\alpha_{-}\left(r\right)}{{\left(\omega -\mu \left(r\right)-\omega_{L}\right)}^{2}}\right], \end{array}$$

where

(18) $$\alpha_{\pm }=\frac{3\gamma (r)}{4}P\left(r\right)\pm \left(\omega \pm \mu \left(r\right)-\omega_{L}\right)Q\left(r\right),$$

(19) $$\begin{array}{ccc} I_{0}\left(r\right) & = & {\left[(\omega^{2}|d|)/(c^{2}\left|r|\right)\right]}^{2},\;P\left(r\right)=\frac{2\Omega^{2}\left(r\right)-\gamma^{2}\left(r\right)}{2\Omega^{2}\left(r\right)+\gamma^{2}\left(r\right)}, \end{array}$$

(20) $$\begin{array}{ccc} Q(r) & = & \frac{\gamma (r)}{4\mu (r)}\frac{10\Omega^{2}\left(r\right)-\gamma^{2}\left(r\right)}{2\Omega^{2}\left(r\right)+\gamma^{2}\left(r\right)},\;\mu \left(r\right)={\left(\Omega^{2}\left(r\right)-\frac{\gamma^{2}\left(r\right)}{16}\right)}^{1/2}. \end{array}$$

It can be seen from Equation (17) that the resonance fluorescence of a two-level QE in free space consists, as a rule, of four components, whose intensities largely depend on the intensity of the controlling field. The decay rates in this equation determine the width of the corresponding Lorentzian lines in the spectrum, which consist of Lorentzian profiles at frequencies $\omega_{L}$, $\omega_{L}\pm \Omega_{\Lambda }$ and a coherent response at the frequency $\omega_{L}$.

In order to calculate the resonance fluorescence near an NP, it is necessary to take into account that the Rabi frequencies and the rates of the radiative decay change. The modified Rabi frequency in the case of a nanosphere is determined by the field components calculated by Equation (3) and has the form

(21) $$\begin{array}{ccc} \Omega (r) & = & \frac{d}{\hbar }\sqrt{|E_{r}{|}^{2}+{\left|E_{\theta }\right|}^{2}}\\ & & =\frac{dE_{0}}{\hbar }\sqrt{{\left|\cos\theta \left(\frac{2a^{3}}{r^{3}}\frac{\varepsilon (\omega )-1}{\varepsilon (\omega )+2}+1\right)\right|}^{2}+{\left|\sin\theta \left(\frac{a^{3}}{r^{3}}\frac{\varepsilon (\omega )-1}{\varepsilon (\omega )+2}-1\right)\right|}^{2}}, \end{array}$$

where *d* is the dipole moment of the QE (in the Gaussian system of units), $E_{0}$ is the amplitude of the external field, ${\hat{n}}_{r}$ and ${\hat{n}}_{\theta }$ are the unit vectors in the spherical coordinate system, and ε(ω) is the dielectric permittivity of the NP (in the case of a sphere $E_{\phi }=0$).

The total normalized decay rate of a QE that is located at point r and has a dipole moment oriented along the direction of the local field at this point can be defined as

(22) $$\gamma /\gamma_{0}=\frac{|E_{r}{|}^{2}{\left(\gamma /\gamma_{0}\right)}_{\mathrm{rad}}+\left(\right|E_{\theta }{|}^{2}+|E_{\varphi }{|}^{2}){\left(\gamma /\gamma_{0}\right)}_{\tan}}{\sqrt{|E_{r}{|}^{2}+|E_{\theta }{|}^{2}+{\left|E_{\varphi }\right|}^{2}}},$$

where ${\left(\gamma /\gamma_{0}\right)}_{\mathrm{rad}}$ and ${\left(\gamma /\gamma_{0}\right)}_{\tan}$ are the total decay rates for the radial and for the tangential orientations of the dipole moment of the QE.

**Figure 7:**
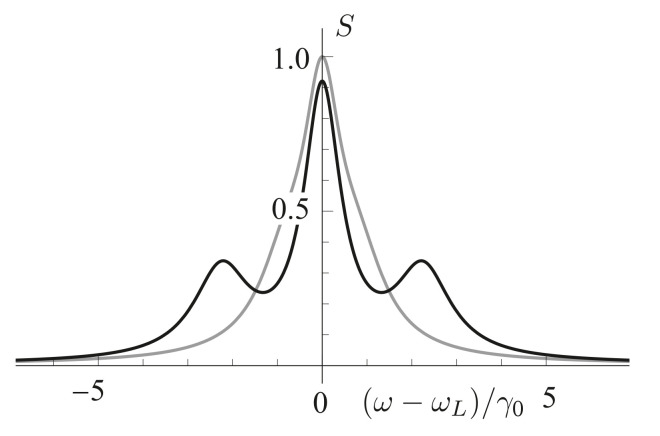
Resonant fluorescence spectra of a two-level QE with $\gamma_{0}=20$ MHz, in free space (gray line) and near a nanosphere (black line) with a radius of a=20 nm at a distance of 20 nm from the nanosphere surface and θ=0.3 rad (17${}^{\circ }$) in a weak laser field. λ=632.8 nm, $E_{0}=500$ V/m.

The results of calculation of the resonance fluorescence spectra of a QE in free space and near an NP are shown in Figure 7. It can be seen from this figure that, in the limit of a weak incident laser field, the resonance fluorescence spectrum of a two-level QE in free space has only one pronounced line in the spectrum at zero frequency detuning. Placing the NP in close proximity to the QE enhances the local field and affects the Rabi frequency and the QE decay rate, so that the resonance fluorescence spectrum becomes enriched, and the triplet structure of the Apanasevich–Mollow spectrum can be clearly seen. Taking into account the effect of these parameters opens up new possibilities for controlling the properties of an atom using NPs.

**Figure 8:**
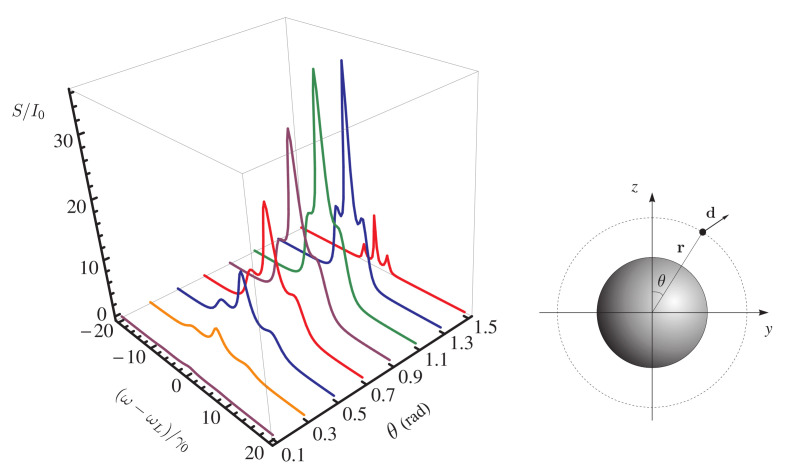
Resonance fluorescence spectra (normalized to $I_{0}$) of a two-level QE near a nanosphere with a radius of a=20 nm in relation to the angle θ = 0.1–1.5 rad ($5.7^{{}^{\circ}}$ to $85.9^{{}^{\circ}}$; the distance from the QE to the nanosphere surface is 10 nm, λ=632.8 nm and $\gamma_{0}=20$ MHz, $E_{0}=2000$ V/m.

Figure 8 shows the behavior of the resonance fluorescence spectrum of a QE located at a distance of 10 nm from the surface of a nanosphere in relation to the angle of arrangement θ of the QE. The spectrum depends on the position of the observer as $S∼\psi^{2}$ (17), ψ is the angle between the direction of the QE dipole and the *z* axis. In this case, the observer is located on the *z* axis, and the dipole moment of the QE is codirectional with the direction of the local field, so that, at the angles $\theta =0^{\circ }$ (0 rad) and $90^{\circ }$ (1.57 rad), the intensity of the resonance fluorescence of the QE that the observer registers at these spatial points tends to zero. The strength of the local field attains a maximum at $\theta =0^{\circ }$ and a minimum at $90^{\circ }$, so that, as θ increases, the side lines of the spectrum shift towards the central line due to a decrease in the Rabi frequency. Note also that, at θ=1.1 rad ($67^{\circ }$), a maximum intensity of the resonance fluorescence is observed (Figure 8).

**Figure 9:**
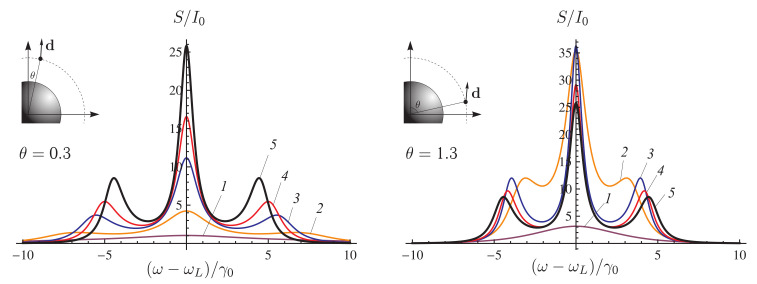
Resonance fluorescence spectra (normalized to $I_{0}$) of a two-level QE located near a nanosphere with a radius of a=20 nm for two positions of the QE: θ=0.3 rad (**left**) and θ=1.3 rad (**right**). The QE–NP distance from the surface is 5 nm (curve 1), 10 nm (2), 20 nm (3), and 30 nm (4). Curve 5 refers to QE in free space. $E_{0}=2000$ V/m, λ=632.8 nm, $\gamma_{0}=20$ MHz.

The resonance fluorescence spectrum of a two-level QE depends significantly on the distance between the QE and the NP surface at fixed values of the angle θ (Figure 9). A decrease in the distance between the QE and the NP surface leads to a narrowing of the resonance fluorescence lines and a decrease in the spacing between them. At distances shorter than 5 nm, the rate of nonradiative decay of the QE significantly prevails over the rate of radiative decay and, as a consequence, the effect of fluorescence quenching is observed.

The above described approach to the analysis of the interaction of a QE with an NP was largely based on the density matrix approximation. A deeper approach to describing the quantum properties of light emitted by a QE in the presence of an NP is based on the application of quantum electrodynamics. Thus, the authors of works [69,127], using quantum electrodynamics, considered the strong resonant interaction of a two-level QE (atom) with a continuum of quantized electromagnetic modes falling into the contour of the resonant mode of a dielectric microsphere and derived analytical solutions. In [128] the effect of quantum fluctuations and correlations on the dynamics of an NP and a two-level QE in an external optical field was taken into account in the approximation of a small number of plasmons. It was shown that, with an increase in the coupling constant between the QE and NP, due to the appearance of the Fano resonance, the shape of the Apanasevich–Mollow triplet initially becomes asymmetric, then the lateral maxima disappear, and then the triplet degenerates into a single Lorentzian line.

It is also important to appreciate the fields of applicability of the quantum and semiclassical descriptions of the systems under consideration. In [129], the fluorescence spectra of a molecule near a plasmonic NP have been studied quantum-mechanically in detail. The authors of that work considered the modes of both weak and strong coupling, as well as the excitation of high-order modes in NPs, and showed that a strong coupling arises at distances shorter than 5 nm and requires a completely quantum-mechanical description, while, to describe interactions at large distances, the semi-classical approximation operates well.

A model of a two-level QE is the simplest one; however, studies of more complex QEs were described in the literature. For example, in [130] the resonance fluorescence of a three-level QE of the Λ-type located near a spherical metallic NP was investigated. The authors considered the case when the QE is excited by a laser field along one of the optical transitions. It was shown that the shape of the spectrum depends on the mutual orientation of the optical transition dipole moments with respect to the surface of the metallic NP. It was also shown that the location and the width of the spectral peaks are strongly modified by the exciton–plasmon coupling and the laser detuning, which makes it possible to obtain a controlled spectral line at the second uncontrolled transition in a system with a width that is significantly narrower than the natural width.

In [131], the resonance fluorescence of a four-level double V-type QE located near a plasmonic NP has been studied theoretically. The quantum system interacted with two orthogonal circularly polarized laser fields with the same frequency and intensity, but with different phases. A two-dimensional array of dielectric nanospheres with a metal coating was considered as a plasmonic NP. This QE demonstrated spontaneous emission quantum interference near the plasmonic NP. The authors of that work showed that the presence of a plasmonic NP leads to a strong modification of the resonance fluorescence spectrum. In addition, the spectrum of its resonance fluorescence and the second-order correlation function strongly depend on the phase, so that the relative phase of the laser fields can be used to effectively control the characteristics of the resonant fluorescence.

There are works whose authors investigate the modification of the resonance fluorescence spectra for various NPs [132,133,134,135,136,137,138]. However, all of these works are theoretical, since the observation of resonance fluorescence of the QE + NP systems is a difficult experimental problem even taking into account the modern development of technologies. Among them, we can single out work [139], which considers an artificial atom, an interesting and promising object for quantum computations. A Josephson qubit was considered as an artificial atom. In that work, the resonance fluorescence of such an atom was experimentally investigated.

### 4.2. Statistics of Photons of the Resonance Fluorescence Spectrum of a QE

An important characteristic of resonance fluorescence is the statistics of the distribution of photocounts of fluorescent photons, which is affected by the occurrence of an NP near a QE. In [140], the statistics of the number of photons of resonance fluorescence of a two-level QE in the vicinity of a metallic spherical NP excited by a laser field with a finite bandwidth was investigated theoretically for the first time. An analysis showed that all the interesting physics here takes place in a small region around the NP, where the near field and the QE–NP coupling significantly affect the emissive properties of the QE. Estimates showed that the size of this region is r≤2a (*r* is the distance from the center of the NP to the QE), and *a* is the radius of the nanosphere. At large distances, the influence of the NP disappears and the QE behaves in the same way as in free space.

**Figure 10:**
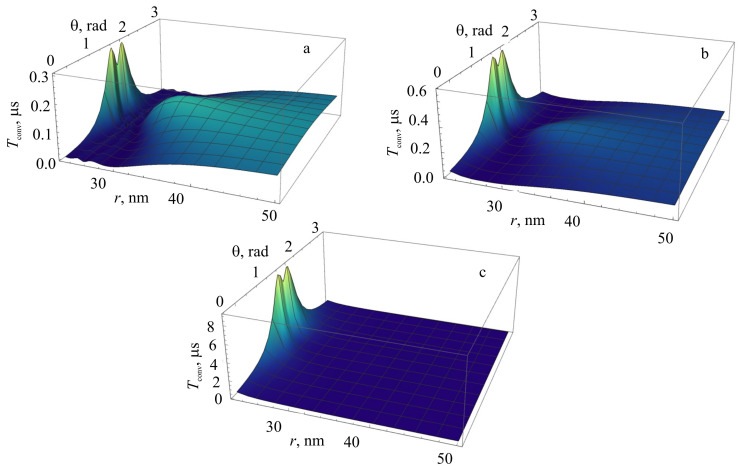
Dependences of the convergence time $T_{\mathrm{conv}}$ on coordinates of a QE *r* (left) and θ (right) around an NP and on the normalized frequency detuning D=0 (**a**), D=1 (**b**) and D=5 (**c**) at $\Delta \omega_{L}=1$ MHz.

Figure 10 shows the behavior of time $T_{\mathrm{conv}}$ of convergence of the photocount statistics to the Gaussian distribution for a QE at a point with coordinates *r*, θ around an NP for three values of detuning *D* of the laser radiation and a finite laser bandwidth $\Delta \omega_{L}=1$ MHz. It follows from the figure that the convergence time significantly depends on the coordinates of the QE around the nanosphere at distances r≤50 nm for D=0, 40 nm for D=1 and 30 nm for D= 5, which essentially reflects the region in which the near field of the metallic NP is significant. Near θ=π/2, the convergence time reaches its global maximum at r=23 nm: $T_{\mathrm{conv}}^{\max}=0.3$ μs for D=0 to $T_{\mathrm{conv}}^{\max}=9$ μs at D= 5, which is two orders of magnitude longer than for QE in free space, for which $T_{\mathrm{conv}}=0.1\;\mu$s. The typical scale of the convergence time at large distances r>2a tends to the convergence time for free space. There are also two regions symmetric with respect to the NP, in which Ω∼γ and the convergence time here tends to zero. This behavior is determined by the interaction of the radiative and nonradiative decay rates of the QE due to the interaction with the metallic NP and the near field.

**Figure 11:**
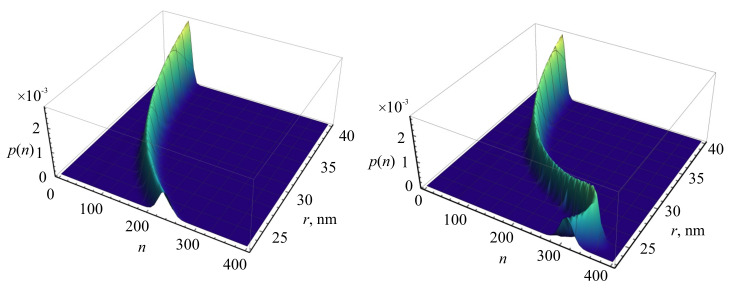
Probabilities p(n,T) in relation to the coordinate *r* of a QE with respect to an NP for the cases of an ideal spherical NP with ε=−∞ (**left**) and for a silver NP with ε=−15.37+i0.231 (**right**) at θ=π/6 rad for T=9 μs, D=0, λ=632.8 nm and $\Delta \omega_{L}=1$ MHz.

The probability distribution function p(n,T) of the emission of *n* photons of the resonance fluorescence of a QE in a given time interval *T* significantly depends on the position of the QE around the NP (Figure 11), demonstrating a characteristic bend in a comb-like dependence. To elucidate the reason for this bend, the probability p(n) was calculated for the fixed parameters D=0, θ=π/6 for different materials, such as a nanosphere made of an ideal metal (Figure 11, left), and of silver (Figure 11, right). It is clearly seen from this figure that the bend of the comb-like dependence p(n) is caused by the nonradiative part of the decay rate of the QE associated with the metallic NP, which is significant in the range 23≤r≤40 nm.

### 4.3. Antibunching of Resonance Fluorescence Photons of a QE

The phenomenon of photon antibunching [141] was theoretically predicted in [126,142] for the fluorescent radiation of a two-level QE that was excited in free space by a resonant laser field and was confirmed experimentally in [3]. The essence of the phenomenon is that there are states of the electromagnetic field for which $g^{2}\left(0\right)<g^{2}\left(\tau \right)$, where $g^{2}\left(\tau \right)$ is the normalized second-order correlation function,

(23) $$g^{2}\left(r,\tau \right)=\frac{\langle {\hat{E}}^{-}\left(r,t\right){\hat{E}}^{-}\left(r,t+\tau \right){\hat{E}}^{+}\left(r,t+\tau \right){\hat{E}}^{+}\left(r,t\right)\rangle }{\langle {\hat{E}}^{-}\left(r,t\right){\hat{E}}^{+}\left(r,t\right)\rangle \langle {\hat{E}}^{-}\left(r,t+\tau \right){\hat{E}}^{+}\left(r,t+\tau \right)\rangle },$$

while the field is considered to be statistically stationary, independent of the initial state of the QE, i.e., t→∞.

The function $g^{2}\left(r,\tau \right)$ is proportional to the probability to detect two photons at a point with coordinate r within time interval τ [143]. Therefore, the phenomenon of photon antibunching is a nonclassical phenomenon, in which photon pairs with a time interval τ≠0 are recorded more often than pairs with a zero time interval.

In the case of a stationary field, the normalized second-order correlation function has the form

(24) $$g^{2}\left(r,\tau \right)=\frac{\left[{\langle {\hat{R}}_{3}\left(r,\tau \right)\rangle }_{G}+\frac{1}{2}\right]}{\left[{\langle {\hat{R}}_{3}\left(r,t\right)\rangle }_{ss}+\frac{1}{2}\right]},$$

where index *G* corresponds to the fluorescent radiation of the QE that initially resided at the lowest energy state, and index ss corresponds to the stationary value (t→∞) of the Pauli operator

(25) $$\langle {\hat{R}}_{3}\left(r,\tau \right)\rangle =\frac{\frac{1}{4}\Omega^{2}\left(1+\frac{\Delta \omega_{L}}{\beta }\right)}{\frac{1}{2}\Omega^{2}\left(1+\frac{\Delta \omega_{L}}{\beta }\right)+{\left(\beta +\Delta \omega_{L}\right)}^{2}+\beta^{2}D^{2}},$$

where β=γ/2, $D=(\omega_{L}-\omega_{0})/\beta$. Therefore, $g^{2}\left(r,\tau \right)$ depends on the local field strength, the spontaneous relaxation rate of the QE (radiative and nonradiative), the laser radiation linewidth, and the detuning from the resonance at the given point. Consequently, $g^{2}\left(r,\tau \right)$ depends on the position of the QE with respect to the nanoantenna.

**Figure 12:**
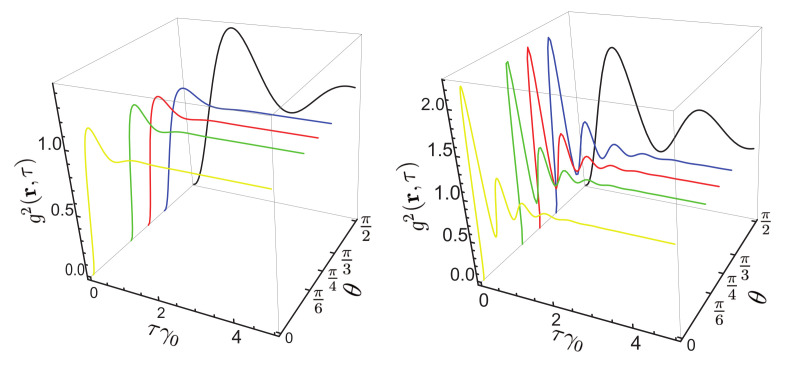
Second-order correlation functions $g^{2}\left(r,\tau \right)$ for different values of angle θ for the distance from the surface of 10 nm in the case of a zero detuning from resonance. The laser radiation (Lorentzian profile) linewidth is $\Delta \omega_{L}=0$ MHz (**left**), $\Delta \omega_{L}=1$ MHz (**right**).

Figure 12 presents the correlation function $g^{2}\left(r,\tau \right)$ for different values of angle θ for the distance to the surface of 10 nm. It can be seen that the correlation function depends on the laser linewidth, and this parameter cannot be neglected (in the following we assume that $\Delta \omega_{L}=1$ MHz).

**Figure 13:**
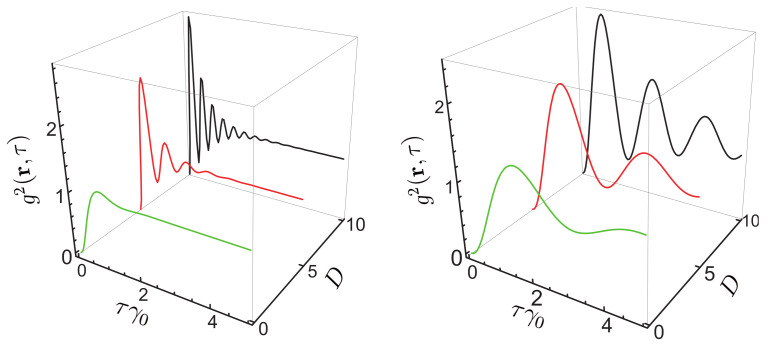
Second-order correlation functions $g^{2}\left(r,\tau \right)$ for different values of angle θ at r=30 nm and different values of the detuning from resonance *D* (the laser profile is the Lorenzian on). The plots from the left refer to θ=π/4, and the plots from the right correspond to θ=π/2.

Figure 13 presents the correlation function for two values of angle θ at r=30 nm for different values of the detuning from the resonance. It can be seen that $g^{2}\left(r,\tau \right)$ depends on the detuning of the transition frequency of the QE from the resonance. The NP affects the transition frequency. Compared to the value of the transition frequency, this change is many orders of magnitude smaller, but the expression for $g^{2}\left(r,\tau \right)$, as well as for the spectral density, depends not on the frequency difference but rather on the ratio $(\omega_{L}-\omega_{0}^{{}^{\prime }})/\beta$, where 2β=γ is the spontaneous relaxation rate of the QE (radiative and nonradiative). This ratio is not negligibly small even for the detuning from the resonance of a few MHz.

The antibunching of fluorescent photons in the case of a three-level QE of the Λ-type that was located near a spherical metallic NP have been investigated in [130]. The authors considered the case when the QE was excited by a single laser field along one of the optical transitions. It was shown in that work that strong antibunching of fluorescent photons is achieved along the second uncontrollable transition in the Λ-system.

In [131], the authors showed that the presence of a plasmonic NP leads as to a strong modification of the resonance fluorescence spectrum, as to transition from antibunching to bunching for fluorescent photons. In [144], the same authors investigated the statistics of the emission of a two-level QE near the same two-dimensional array of dielectric nanospheres with a metal coating. It was shown that the photon statistics is significantly modified by a lattice of NPs. The transition time from antibunching to bunching depends very strongly on the orientation of the QE relative to the lattice, on the transition frequency of the QE, on the strength of the applied electric field, and on the geometric parameters of the NP. In particular, for a weakly excited QE, whose dipole moment is parallel to the plasmonic lattice, the characteristic time of the transition from antibunching to bunching of photons can increase by two orders of magnitude in comparison with the case of free space. Under stronger external excitation, when the external Rabi frequency is fixed, a decrease (or increase) in the transition time from antibunching to bunching of photons is observed, when the decay rates decrease (or increase) with respect to their values in vacuum. In addition, the thickness of plasmonic nanoshells can act as a separate controlling parameter of the photon statistics.

In the works considered above, we were dealing with single QEs. Now, there is an increasing interest in the study of the properties of light generated by hybrid systems, including a mesoscopic amount of QEs to create macroscopic quantum light sources. Thus, in [145], the statistical properties of light generated by a set of QEs associated with one and the same electromagnetic mode were investigated. Theoretical calculations based on the calculation of the effective Hamiltonian of the system were performed to describe the response of two different systems under low-intensity coherent excitation of plasmonic nanocavities and dielectric microcavities.

The results of that work showed that there are two different mechanisms that lead to significant negative correlations in the interaction between the purely bosonic (cavity) and quasi-bosonic (ensemble of QEs) subsystems: photonic blockade and destructive interference. The first occurs at a high coupling strength (comparable to or exceeding the rate of radiation emission from the cavity), while the second becomes relevant at weaker cavity +QE interactions.

When considering QE + NP systems, the quantum properties of the QE radiation are usually studied, while the plasmonic NP is considered as a passive element that changes the local environment of the QE. However, in the case of a strong coupling between the NP and QE, the NP itself can emit light with quantum properties. Unlike the situation described above, the metallic NP itself can emit light. Upon optical excitation above the onset of interband transitions, noble metal NPs can exhibit rather strong visible photoluminescence. There is experimental evidence that the nonradiative relaxation channel in this case can include the generation of localized surface plasmons (LSPs), which subsequently emit light. Coupling of a metallic NP with a QE can lead to enhancement of photoluminescence of the metallic NP. At the same time, LSPs can be excited directly either by fast electrons or by light resonant to the LSP mode. The emission rate of a dipole LSP in a metallic NP is proportional to its volume and can reach values on the order of $10^{3}$ THz, which is three orders of magnitude higher than the limiting rate expected for quantum effects associated with metallic nanostructures.

The problem of studying the quantum properties of light emitted by a system of a metallic NP strongly coupled with a QE was considered in [146]. It was shown that such a system behaves effectively as a two-level system and should spontaneously emit light that obeys the sub-Poissonian statistics and exhibits ideal antibunching. Two different scenarios of excitation were considered in that work: (i) a metallic NP is excited nonresonantly through the interaction with a three-level QE and (ii) the metallic NP + QE system is excited resonantly well below the saturation. It was found that, in the first case, the rate of one-photon generation is fundamentally limited by the rate of nonradiative relaxation in the excitation channel. On the contrary, the latter scenario is considered as a simple and promising approach that can provide a repetition rate of single-photon radiation on the order of 100 THz and can be used to create single-photon radiation sources.

In real experiments on resonance fluorescence, spectral filtering of resonance fluorescence is widely used to improve the purity and indistinguishability of single photons by removing unwanted background. The use of this technique should certainly affect the radiation statistics. It was shown in [147] that the resonance fluorescence spectrum of a two-level QE contains many lines, each of which demonstrates different photon statistics. Without filtering, these components always interfere with the strong antibunching expected from single QEs. However, with spectral filtering whose bandwidth is comparable with the natural linewidth or the Rabi frequency, the ratio of these components changes in the filtered spectrum, which leads to strongly altered photon statistics. For weak resonant excitation, an appropriate narrow filter removes almost all of the incoherent components, destroying antibunching and demonstrating that the subnatural linewidth and strong antibunching cannot be measured simultaneously. Under strong resonant excitation, a noticeable bunching effect is observed at filter passbands comparable to the natural linewidth before the system eventually begins to tend towards the Poissonian statistics for the narrowest filters. These results illustrate a potentially new approach to controlling the statistics of photons in quantum light. Thus, when filtering the QE spectrum, care must be taken to maintain antibunching.

### 4.4. Formation of Squeezed States of Light

We will now proceed to the consideration of the phenomenon of radiation squeezing [141], which is a purely quantum phenomenon, and we will show that an NP can lead to the squeezing of fluorescent radiation of a two-level QE located in the near field of an NP.

Let us consider the quadrature squeezing of the fluorescent radiation field. Let ${\hat{E}}_{1}={\hat{E}}^{+}+{\hat{E}}^{-}$ and ${\hat{E}}_{2}=-i\left({\hat{E}}^{+}-{\hat{E}}^{-}\right)$ be the Hermitian operators for two components of the field strength vector that differ by π/2 in phase. In this section, for simplicity, the field operators will be considered to be normalized to $\omega^{2}d/(4\pi \varepsilon_{0}c^{2}|r-r_{0}\left|\right)$. In addition, let us assume that, for each position of the QE, the detector is in the far-field zone perpendicularly to the QE dipole moment; i.e., sinη=π/2. Let

$$[{\hat{E}}^{+},{\hat{E}}^{-}]=C,$$

where *C* is the function of *r* and θ, which also depends on the laser radiation spectral width and on the radiation detuning from the resonance of the QE. Then the corresponding commutator of the operators ${\hat{E}}_{1}$ and ${\hat{E}}_{2}$ equal to:

$$[{\hat{E}}_{1},{\hat{E}}_{2}]=2iC.$$

Therefore, the condition of squeezing in our case is expressed as ${\hat{E}}_{1}$ and ${\hat{E}}_{2}$ is, correspondingly, equal to [148]:

$$\langle {\left(\Delta {\hat{E}}_{1}\right)}^{2}\rangle =C+\langle :{\left(\Delta {\hat{E}}_{1}\right)}^{2}:\rangle ,\;\langle {\left(\Delta {\hat{E}}_{2}\right)}^{2}\rangle =C+\langle :{\left(\Delta {\hat{E}}_{2}\right)}^{2}:\rangle ,$$

where “:” is the normal ordering.

Therefore, the condition of squeezing in our case is expressed as $\langle :{\left(\Delta {\hat{E}}_{1}\right)}^{2}:\rangle <0$ or $\langle :{\left(\Delta {\hat{E}}_{2}\right)}^{2}:\rangle <0$. It should be noted that there is no classical analog for the squeezed state. Therefore, the conditions of squeezing for the components ${\hat{E}}_{1}$ and ${\hat{E}}_{2}$, respectively, take the form

(26) $$\langle :{\left(\Delta {\hat{E}}_{1}\right)}^{2}:\rangle =2\langle {\hat{E}}^{-}{\hat{E}}^{+}\rangle -{\left(\langle {\hat{E}}^{+}\rangle +\langle {\hat{E}}^{-}\rangle \right)}^{2}<0,$$

(27) $$\langle :{\left(\Delta {\hat{E}}_{2}\right)}^{2}:\rangle =2\langle {\hat{E}}^{-}{\hat{E}}^{+}\rangle -{\left(\langle {\hat{E}}^{+}\rangle -\langle {\hat{E}}^{-}\rangle \right)}^{2}<0.$$

In [149], the squeezing of fluorescent radiation was considered; however, the linewidth of the exciting field was not taken into account.

**Figure 14:**
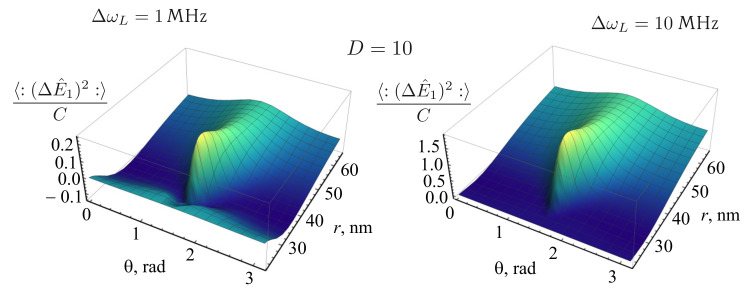
Dependences of quantity $\langle :{\left(\Delta {\hat{E}}_{1}\right)}^{2}:\rangle$ normalized to *C* on the position of a QE at D=10 and different values of $\Delta \omega_{L}$: $\Delta \omega_{L}=1$ MHz (**left**) and $\Delta \omega_{L}=10$ (**right**) in the presence of a nanosphere.

Figure 14 shows the plots of the dependences of the $\langle :{\left(\Delta {\hat{E}}_{1}\right)}^{2}:\rangle$ normalized to *C* on the position of the QE in the vicinity of a nanosphere at D=10 for different values of $\Delta \omega_{L}$: $\Delta \omega_{L}=1$ MHz (left) and $\Delta \omega_{L}=10$ MHz (right) in the presence of the nanosphere. It can be seen that, with an increase in the laser linewidth, the effect of the radiation squeezing for the field component ${\hat{E}}_{1}$ disappears.

**Figure 15:**
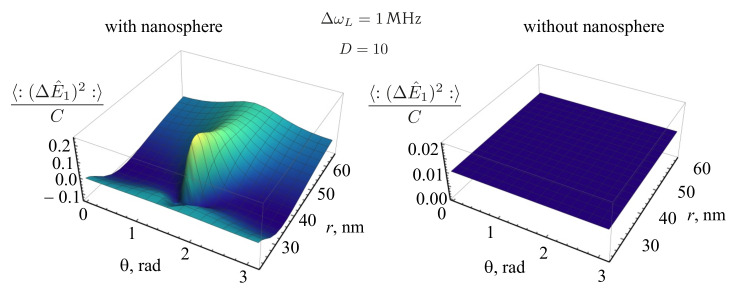
Dependences of quantity $\langle :{\left(\Delta {\hat{E}}_{1}\right)}^{2}:\rangle$ normalized to *C* on the position of a QE at D=10 in the presence of a nanosphere (**left**) and in its absence (**right**).

Figure 15 shows the dependences of the $\langle :{\left(\Delta {\hat{E}}_{1}\right)}^{2}:\rangle$ normalized to *C* for the case of a QE in the presence (left) and in the absence (right) of a nanosphere for the value of the detuning D=10. It can be seen from the figure that, in the absence of the nanosphere, for the given parameters of the incident radiation, the fluorescent radiation is unsqueezed. Squeezing occurs due to the presence of the nanosphere, near its surface. The squeezing also depends in a complex way on the Rabi frequency Ω and on the spontaneous relaxation rate γ, which, in turn, depend on the position of the QE with respect to the nanosphere.

There are also works that consider various quantum effects in systems QE + NPs. In particular, the effects of quantum coherence and interference in a QE near an NP were studied in [150]. In that work, the formation of dark states, optical pumping, coherent Raman scattering and STIRAP (Stimulated Raman Adiabatic Passage) in the presence of metallic NPs were demonstrated. It was shown that dark states are formed, but they have more complex structures in the presence of an NP, whereas optical pumping and STIRAP cannot be used near an NP. The STIRAP method should be used with care because it may not work or, at least, may have new functions in the presence of an NP.

In [151], a system consisting of metallic NPs and a set of QEs was considered. If the coupling between the QE and NP is weak, the well known phenomenon of fluorescence quenching arises; in this regime, the electromagnetic modes are considered as a quasi-continuum. It was shown in that work that the concept of quenching is violated if a strong coupling arises between QEs and electromagnetic modes of the NP. In this limit, higher-order multipole modes can no longer be regarded as a quasi-continuum, and the description using pseudo-modes becomes more appropriate. Using numerical and analytical simulations, the authors demonstrated that the quenching pattern in the case of a weak coupling is strongly transformed upon passing to the strong coupling regime, whereas, in the weak-coupling limit, nonradiative electromagnetic modes of NPs act as an effective thermostat for the radiation of QEs, upon passage to the strong-coupling regime, they become a pseudo-mode capable of reversibly exchanging energy with QEs. This energy transfer occurs between each emitter and the localized pseudo-mode and is, therefore, not a cooperative effect. However, as the number of QEs increases, the collective strong coupling of many QEs to the radiative dipole mode of NPs grows even at very short distances.

An obvious development is the consideration of increasingly complex nanostructures. For example, in [152], the interaction between QDs and core–double shell NPs was theoretically investigated within the completely quantum approach. The possibility of controlling the spontaneous emission of QDs located near such particles and inside them (in shells) was considered. It was shown that NPs of the core–double shell type, as compared to simple NPs without shells, can switch the absorption saturation between NPs and QDs, with this effect being especially pronounced if QDs are placed inside the shell. Such systems are of interest for creating quantum plasmonic sensors.

The works that consider NPs made of special materials are also of interest. For example, in [153], the optical properties of a dimer that is formed by an NP based on a topological insulator (NTI) and a QE, which interact in a strong coupling regime, were investigated theoretically. Topologically protected (surface) states affect the optical properties of an NP based on a topological insulator, demonstrating that, under the action of light, a single electron in such a state creates a surface charge density similar to a plasmon in a metallic NP. In addition, such an electron can act as a screening layer, effectively suppressing absorption inside the NP, and can couple phonons and light, inducing a topological polariton mode (TPM) of the NP, which has not been registered before. In that work, the authors theoretically investigated the behavior of this mode in the case when the NTI strongly interacts with a single QE by calculating the spectrum of the system. In particular, it was shown that the TPM is strongly coupled to the QE resonance, causing a hybrid mode, whose output signal, as well as its spectral position, can primarily be tuned by controlling the size of the NTI and the distance between the NP and QE.

### 4.5. Generation of Entangled Quantum States

For applications that use quantum entangled states, it is desirable that these states, once created, would exist for a sufficiently long time [154,155,156]. However, the results of many works show that dissipative processes in the system and the decoherence associated with them are a serious obstacle to the preservation of entanglement. However, this statement turns out to be erroneous when the entanglement is generated by linking qubits with a common dissipative medium. These schemes are extremely attractive from the practical point of view because they allow one to achieve entanglement irrespective of the initial state of the system and, as has been shown, are rather stable to changes in controlling parameters. Metallic NPs can act as such dissipative media.

Initially, metal nanowires were considered as such nanostructures. Surface plasmon polaritons are excited in them and propagate along the nanowires. In this geometry, quantum states of qubits, where quantum dots, atoms, or even molecules act as qubits, can be entangled if brought close to the nanowire. For identical qubits, the maximum degree of entanglement can be obtained by choosing an appropriate relative coupling strength with the nanowire. In such a scheme, multiparticle entanglement between qubits and plasmons is achieved, followed by a measurement of the amount of plasmon excitations at the ends of the nanowire. Only if plasmons are not detected can we conclude that the qubits were most entangled with each other.

In order to obviate this probabilistic nature of obtaining entangled states and to create a high degree of entanglement in deterministic schemes, it was proposed to asymmetrically arrange the qubits with respect to the nanowire. At certain values of the coupling strength, the maximum degree of entanglement can be achieved irrespective of the state of the plasmonic field [157,158]. However, large losses associated with the propagation of surface plasmons do not allow maintaining entanglement for a long period of time.

The idea of using loss channels to convert qubits to a subradiant state that is resistant to dissipation was studied in [159]. The described scenario makes it possible to achieve stationary entanglement. This requires that the system was initially brought into a predetermined state, and, moreover, the resulting entanglement would remain well below the maximum possible.

Recently, a robust-to-loss entanglement generation scheme with a waveguide consisting of an array of metallic NPs was also been proposed [160], whereas nanoscale optical lattices were proposed to control the interaction of distant ultracold atoms through their interaction with collective plasmonic modes [161].

However, none of the schemes described above can provide a maximum stationary entanglement of a pair of qubits in a deterministic way. To achieve it, it was proposed to use strongly dissipative systems.

In contrast to works in which the propagation of surface plasmon polaritons in metal waveguides was considered, there are works whose authors considered an isolated metal nanoantenna as a dissipative structure. Thus, in [162], a scheme was proposed for creating a maximally entangled state between two qubits due to a dissipatively controlled process. For this purpose, the quantum states of the qubits are entangled, which are mutually connected by the plasmonic nanoantenna. When a weak spectral asymmetry is imposed in the properties of qubits, the steady-state probability of obtaining maximally entangled subradiant state approaches unity. This occurs despite the high losses associated with the plasmonic nanoantenna, which are generally considered harmful. The authors showed that the entanglement scheme is quite robust against variations in the transition frequencies of quantum dots and deviations in their prescribed position relative to the nanoantenna, and the maximum entanglement can be obtained using a symmetric coupling constant.

A similar system was examined in [163]. In that work, two identical QEs were placed near a spherical metallic NP. The frequency of the dipole plasmon resonance coincided with the frequency of the radiative transition of the QE. A scheme was proposed in which two QEs were resonantly coupled with the dipole plasmon resonance of the NP, and only one QE was initially excited. In such a scheme, it was possible to form a stable coherent superposition state much faster than the spontaneous emission of an isolated QE. In this superposition state, the nonzero dipole moments of the QEs do not coincide in phase with each other, so that the total electric field acting on the NP disappears, thereby eliminating the energy dissipation (that is, the absorption of the field in the NP) and significantly increasing the lifetime of the entangled state. It was shown in that work that the degree of stationary entanglement depends only on the ratio of the distances between the QE and NP, reaching a maximum value of 0.65, when the distance between the NP and the initially excited QE is 20% greater than the distance from the other QE to the NP.

A particular feature of the process under consideration is that, despite the nonzero dissipation in the system (due to the absorption of the NP radiation), the entanglement remains constant for a time much longer than the characteristic time of the system until the much slower process of spontaneous emission begins.

A similar system was considered in [164], which consisted of two qubits with quantum dots coupled with a common damped mode of surface plasmons; however, in contrast to the scheme proposed in [163], each QD is also coupled with a separate mode photon cavity. QDs and plasmons were excited by a 20 fs laser pulse. The QD entanglement, defined as the degree of coincidence according to Wootters, can be detected by measuring the correlation function $g^{\left(2\right)}\left(\tau \right)$ of the cavity photon and can be preserved in high-Q optical cavities.

A similar approach, when the second-order temporal correlation function is used as an indication of the presence of entanglement between a pair of qubits coupled with a common plasmon nanostructure, was considered in [165].

Further development is associated with an increase in the number of QEs and NPs in the systems under consideration. In [166], two, three, and four QDs were considered in the vicinity of a plasmonic nanosystem consisting of metallic NPs; extension to an arbitrary number of QDs was studied in [167]. A system was proposed in which the control of the interaction between entangled QDs and a dissipative medium (plasmonic system) was determined by the nanoscale geometry of the system, and the only requirement for external parameters was an ultrafast laser pulse (single or repetitive).

In contrast to schemes that are based on plasmonic waveguides, which can entangle spatially separated QDs, but are limited to two QDs, the proposed scheme makes it possible to create and maintain entanglement between all pairs of QDs when two, three, etc., closely spaced QDs are coupled with a common plasmonic nanostructure. Moreover, in such a scheme, the entire system is initially in the ground state and is excited by a common laser pulse or a series of pulses.

In all cases, a large degree of coincidence is achieved only for a short period of time after the excitation of the system by a laser pulse, before the population of the entangled state decays. However, entanglement can be restored after applying a repeated series of pulses. A change in the degree of coupling between each QD and a plasmonic nanostructure makes it possible to generate an excited state, which spontaneously develops towards an entangled state because of dissipation of plasmons. Entanglement is achieved without the need for addressing individual QDs.

The dynamics of two qubits located near a two-dimensional lattice of dielectric NPs coated with a metal was considered in [168]. Either qubit locally and independently of the other interacts with the plasmonic nanostructure. The paper considers two different cases: two identical two-level systems and two identical three-level V-type systems, where one two-level transition plays the role of a qubit, while the other level acts as an auxiliary one. A two-dimensional lattice of dielectric NPs coated with a metal was taken as a plasmon nanostructure. The presence of this nanostructure can lead to strongly suppressed rates of spontaneous emission of individual quantum systems, as well as to strongly anisotropic rates of spontaneous decay for orthogonal dipole matrix elements due to the anisotropic Purcell effect, which leads to a quantum interference in the spontaneous emission of radiation. Both effects can be used to dramatically alter the entanglement dynamics of the system.

The majority of works on dissipatively controlled entanglement are limited to two qubits, whereas many-qubit entanglement has been studied very little because of its computational difficulty. In [169], a scheme was considered with a large number of QDs located in one plane around a spherical plasmonic NP. Since the number of qubits is large, the number of combinations of controlling parameters increases exponentially. Therefore, upon modeling multi-qubit entanglement, the controlling parameters are usually set to be symmetric for different qubits. However, previous studies show that the asymmetry of the controlling parameters, especially the asymmetric detuning, plays an important role in a two-qubit system, and, therefore, in the case of multi-qubit entanglement, asymmetric detuning plays an important role. The authors of [169] have proposed an effective model of a multi-qubit system, which makes it possible to reduce the computational complexity of the problem. A scalable plasmonic system consisting of a multi-qubit and a dissipative NP is designed to generate a multi-qubit dissipatively controlled entanglement. Numerical modeling of a plasmonic system shows that the steady-state entanglement caused by a nonzero asymmetric detuning is much greater than that at resonance. In addition, the number of pairs of qubits with antisymmetric detuning plays an important role in increasing entanglement, and maximum entanglement is obtained by appropriately combining controlling parameters. In addition, both the analytical and the numerical calculations showed that a sudden disappearance of entanglement and then its revival occurs when the controlling field is sufficiently large (>5 MeV); with the negativity being chosen as the entanglement criterion [170].

It is important to note that, at the moment, direct experimental observations of entanglement in such systems are an extremely difficult problem; therefore, at the present time, all studies are concentrated mainly in the theoretical field.

## 5. Conclusions

In summary, in this review, we considered the effects of quantum optics of QEs in the near field of nanostructures. The mechanisms by which the rates of radiative and nonradiative decay are modified were described in a simplest model of a two-level QE located near a plasmonic NP, and the intensity and polarization distributions of the near field around the NP were analyzed. These distributions have complex structures, which significantly depend on the polarization of the external radiation field and the parameters of plasmonic resonances of the NP. We also analyzed the effects of quantum optics in the system NP + QE plus external laser field: modification of the resonance fluorescence spectrum of the QE in the near field, bunching/antibunching phenomena and quantum statistics of photons in the spectrum of resonance fluorescence, formation of squeezed states of light, and quantum entangled states in such systems.

Investigations in all these fields are actively developing from year to year, although, due to the difficulty of experiments, the majority of studies are theoretical. The development of nanophotonics and its experimental potential, however, will not stop, and, in the coming years, these studies will increasingly move to the experimental plane.

**Figure 16:**
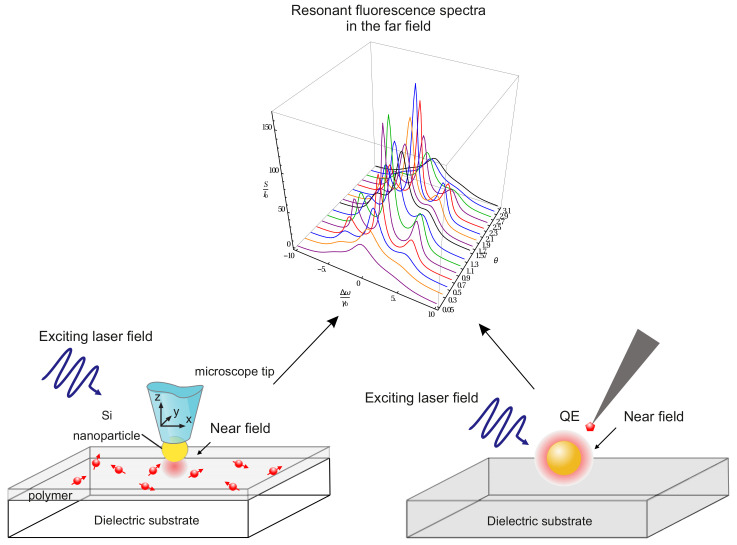
Schemes of two possible experiments on measuring the parameters (intensity and polarization) of the near field of an NP from the registration of the resonance fluorescence spectrum of a QE that interacts with the NP in the far field.

Here, we would like to schematically describe possible experiments to study the properties of the near field of the QE + NP system by measuring and subsequent analyzing the resonance fluorescence spectra of the QE in the far-field zone (Figure 16). In the first of them (Figure 16, left), the near field around the NP can be measured using QEs (fluorescent molecules or QDs) embedded into a polymeric film deposited on a dielectric substrate. The NP is attached to a microscope tip that can be positioned in close proximity to one of the QEs. The QE + NP system is excited by incident laser radiation with the required polarization [82,83]. Then, by measuring the resonance fluorescence spectrum of the QE in the far field, one can obtain information about both the intensity and the polarization of the near field at the point in space where the QE is located [120].

An alternative experiment can be performed with a dielectric NP located either above or on a dielectric substrate, which is realized in the experiment [171,172]: a QE (e.g., a nanodiamond with an NV center) can be incorporated into a tip, which can be placed around an NP with high accuracy [173]. The resonance fluorescence spectrum of this QE measured in the far field will give detailed information about the intensity and the polarization of the near field at the point where the QE is located (Figure 16, right).

A strategy of the 3D polarization mapping that is different from these experiments is as follows. An NP is placed into a viscous liquid that contains fluorescent molecules (QEs), which slowly fall down in the liquid under the gravity (or they rise smoothly under the influence of homogeneous heating). QEs that move downward around the NP are registered in one of the polarization-sensitive schemes using single-molecule nanoscopy, which allows one to accurately determine the orientation of the molecule in space.

We also would like to point out fields of research which are relevant and which will undeniably be actively developed in the near future. Among them is the interaction of structured light with chiral nanostructures [174,175,176,177,178] and dielectric NPs with a high refractive index [179,180,181,182]. We also note experimental studies of interference effects that arise upon interaction of an NP and a QE. One of them is the so-called cloaking effect [172]. It is well known from conventional optics that macroscopic objects cast a shadow in a beam of light, and this shadow becomes darker if the medium is made optically thicker. This scenario also holds true at the nanoscale, when the size of the object is smaller than the wavelength of light. For example, a gold NP with a diameter smaller than 100 nm can extinguish more than half the power of a laser beam if the NP is placed in the focus. According to the Beer–Lambert law, this shadow becomes exponentially darker as a greater number of particles are added. In practice, a metallic NP can be made virtually invisible upon illuminating it with light by placing a QE resonant with the frequency of the incident light in the correct position in the path of the light beam.
